# Pain management in non-operatively treated patients with multiple single rib fractures: an international comparison

**DOI:** 10.1007/s00068-026-03129-0

**Published:** 2026-02-27

**Authors:** Tiemen E. T. Holtrop, Yasmin Arda, George C. Velmahos, Pieta Krijnen, Inger B. Schipper, Joshua S. Ng-Kamstra, Erwin A. Gorter

**Affiliations:** 1 Department of Trauma Surgery, Leiden University Medical Center, Postzone K6-R, P.O. Box 9600, Leiden, 2300 RC The Netherlands; 2 Division of Trauma, Emergency Surgery, and Surgical Critical Care, Department of Surgery, Massachusetts General Hospital and Harvard Medical School, 165 Cambridge St, Suite 810, Boston, MA 02114 USA

**Keywords:** Chest trauma, Rib fractures, Pain management, Opioid, Treatment variation, International comparison

## Abstract

**Purpose:**

Evidence on non-operative management of multiple single rib fractures remains limited, resulting in institutional treatment variation. This study compared outcomes of different pain management strategies.

**Methods:**

A retrospective cohort study at Massachusetts General Hospital (MGH, USA) and Leiden University Medical Center (LUMC, The Netherlands) included patients ≥ 18 years with ≥ 3 single rib fractures between 2018 and 2023. Exclusion criteria included flail chest, surgical fixation, severe extra-thoracic trauma, Glasgow Coma Scale < 15 after 72 h, and transfers. Primary outcomes were pain score change, discharge opioid use, and pulmonary complications. Secondary outcomes included mortality and hospital length of stay (HLOS). Subgroup analysis compared fracture side and displacement, and ICU admission. Forward stepwise logistic regression analysis identified predictors of pneumonia.

**Results:**

545 patients were included (109 at LUMC). LUMC patients were younger (61 vs. 68 years, *p* < 0.001) with fewer comorbidities but higher injury severity scores (13 vs. 10, *p* < 0.001). LUMC favored patient-controlled analgesia (22.9% vs. 5.7%, *p* < 0.001) and less provider-controlled opioids (11.0% vs. 21.3%, *p* < 0.001). Pain score reduction (4 vs. 4, *p* = 0.68), discharge opioid use (45 vs. 45 oral morphine equivalents, *p* = 0.75), HLOS (3 vs. 4 days, *p* = 0.059), pulmonary complications (3.7% vs. 6.2%, *p* = 0.31), and mortality (2.8% vs. 1.8%, *p* = 0.54) were similar. Bilateral and displaced fractures required ICU admission more often but had similar outcomes. Congestive heart failure (OR 14.86, 95%CI 3.24–68.16, *p* = 0.001) and chronic obstructive pulmonary disease (OR 8.08, 95%CI 1.72–38.03, *p* = 0.008) independently predicted pneumonia.

**Conclusion:**

Despite differences in patient characteristics and pain management, outcomes were similar. Hospital level treatment heterogeneity may be acceptable when tailored to patient needs, questioning the need for rigid guidelines.

## Introduction

While consensus guidelines recommend surgical stabilization and rib fixation (SSRF) for flail chest [1, 2], standardized treatment guidelines for multiple single rib fractures, defined as three or more ribs each fractured in only one location, are lacking [3]. This allows for institutional and regional differences in treatment. These fractures are most often treated non-operatively, focusing on adequate pain control, early mobilization, and pulmonary hygiene. Non-operative management aims to reduce mortality and morbidity by enabling coughing, deep breathing, and early mobilization [4]. 

Many different pain management approaches exist, including systemic and regional analgesics, either as standalone treatments or in multimodal strategies, and this variety complicates treatment decisions [5]. Patients with multiple rib fractures may experience an extended hospital stay due to the need for intensive pain management and higher complication rates [6]. An extended hospital stay reflects the clinical complexity and the resource burden caused by these injuries, contributing to higher healthcare costs [7]. While adequate pain control is essential, prolonged opioid use is associated with clinically significant side effects, including respiratory depression, tolerance, and physical dependence [8]. Healthcare systems differ in their opioid prescribing habits. Western-European countries, including The Netherlands, show more conservative opioid usage compared to the United States [9–11]. Also, treatment decisions regarding pain control strategies, use of adjunctive therapies such as regional anesthetics, and discharge planning are often guided by local expertise, resource availability, and institutional preferences. Differences in pain management strategies may influence patient outcomes, including complication rates, opioid use, and hospital length of stay. Comparing treatment approaches between trauma centers could help identify best practices and guide the development of standardized treatment guidelines.

While existing literature on rib fractures predominantly focuses on flail chest injuries [1, 2, 12–14], evidence for the treatment of multiple single rib fractures remains limited [15]. Although some international multicenter studies have examined outcomes in the multiple rib fracture population [16], studies often include mixed populations of flail-chest, segmental, and single rib fractures [15, 17]. Furthermore, these studies have not compared treatment variation between centers or healthcare systems. With healthcare systems known to differ in their pain management approaches [9–11, 18], a cross-institutional comparison could reveal treatment variation in rib fracture care and provide valuable insights into effective pain management strategies for this population.

This study aimed to assess the effectiveness of different pain management strategies and evaluate clinical outcomes in patients with multiple single rib fractures at two level 1 trauma centers: Massachusetts General Hospital (MGH, USA) and the Leiden University Medical Center (LUMC, The Netherlands).

## Methods

### Study design

This retrospective cohort study included patients that were treated at two level 1 trauma centers between 2018 and 2023: Massachusetts General Hospital (MGH) in Boston, Massachusetts, USA, and Leiden University Medical Center (LUMC) in Leiden, The Netherlands. Approval for the study was obtained by the institutional review boards of both MGH and LUMC.

### Participants

Patients 18 years or older who were admitted with multiple single rib fractures (≥ 3 ribs with a single fracture) on CT scan after blunt chest trauma who were treated primarily non-operatively were included. Criteria for exclusion were a radiological flail segment (≥ 3 consecutive ribs fractured in two or more locations), a Glasgow Coma Scale score < 15 at 72 h post-admission, or severe concomitant injuries with an Abbreviated Injury Scale (AIS) ≥ 3 in regions other than the chest, to reduce confounding from non-thoracic injuries. Patients transferred to and from other institutions and patients who underwent immediate SSRF were also excluded. Patients were identified through the trauma registries of both centers using AIS code 450,203 for multiple rib fractures without flail, and then individually reviewed to confirm study eligibility according to inclusion and exclusion criteria.

### Data collection

Data was collected from electronic medical records and stored in Castor EDC, an encrypted online data collection tool. Data collected included patient demographics (age, sex, comorbidities), injury characteristics (rib fracture characteristics, concomitant injuries), initial admission location, pain management (strength of initial pain management, change in management within 72 h, pain scores at admission and discharge, opioid use at discharge), and clinical outcomes. Rib fractures were described according to the taxonomy of the Chest Wall Injury Society, which includes fracture location, type and displacement [19]. Fractures without cortical contact on imaging were categorized as displaced. Pain modalities were ranked into 7 categories from least strong to strongest (no pain medication, oral non-opioid, lidocaine patch, oral opioid, IV opioid, IV patient-controlled analgesia (PCA), nerve block/epidural) and analyzed as an ordinal variable. Non-opioid analgesics primarily included acetaminophen and NSAIDs. Oral opioids commonly used included oxycodone and tramadol. Neither center has formally written protocols specifically for pain management in patients with multiple single rib fractures. Both institutions follow general trauma pain management principles based on the WHO analgesic ladder. Pain management decisions are made based on clinical judgment, considering factors including pain severity scores and patient comorbidities, with treatment individualized to clinical response. Change in pain management strategy was defined as no change, or step-up or step-down in strength of pain management. Opioid use at discharge was documented as Oral Morphine Equivalents (OME) for standardized comparison [20]. 

### Outcomes

The primary outcomes were pain management effectiveness, measured by pain score change between admission and discharge, opioid use at discharge, and pulmonary complications (pneumonia treated with antibiotics, ventilator-associated pneumonia, empyema, in-hospital hemothorax, in-hospital pneumothorax, and unplanned intubation). Pneumonia was defined as antibiotic treatment for suspected pulmonary infection confirmed by imaging, microbiology, or clinical symptoms. Pain scores were recorded on a numeric rating scale (NRS) from 0 (no pain) to 10 (worst imaginable pain). Secondary outcomes included in-hospital mortality, readmission, hospital length of stay (HLOS), and ICU length of stay (ICU LOS).

### Statistical analysis

Counts and percentages summarized categorical variables, and the Pearson’s Chi-squared test was used for univariate group comparisons. Continuous variables were reported as medians and interquartile ranges. Both continuous and ordinal variables were compared between groups using the Mann-Whitney U test. Subgroup analyses included outcomes comparisons between unilateral vs. bilateral fractures, displaced vs. non-displaced fractures, and ICU vs. non-ICU patients. Forward stepwise logistic regression analysis was performed to identify independent predictors of pneumonia. Potential predictors in this analysis were selected based on their assumed biological, mechanical, or clinically plausible risk of pneumonia, and included patient factors (age, sex, obesity, smoking, COPD, asthma, CHF, diabetes, chronic opioid use), medication use (bronchodilators, inhaled corticosteroids, oral corticosteroids), injury characteristics (ISS, number of fractured ribs, fracture laterality, multiregional fractures, fracture type, fracture displacement), and concomitant thoracic injuries (pneumothorax, hemothorax, pulmonary contusion). Entry criteria were set at *p* < 0.05 and removal criteria at *p* > 0.1. Statistical significance was assumed for p-values below 0.05. Statistical analyses were performed using STATA version 16.1 (StataCorp, College Station, TX).

## Results

**Fig. 1:**
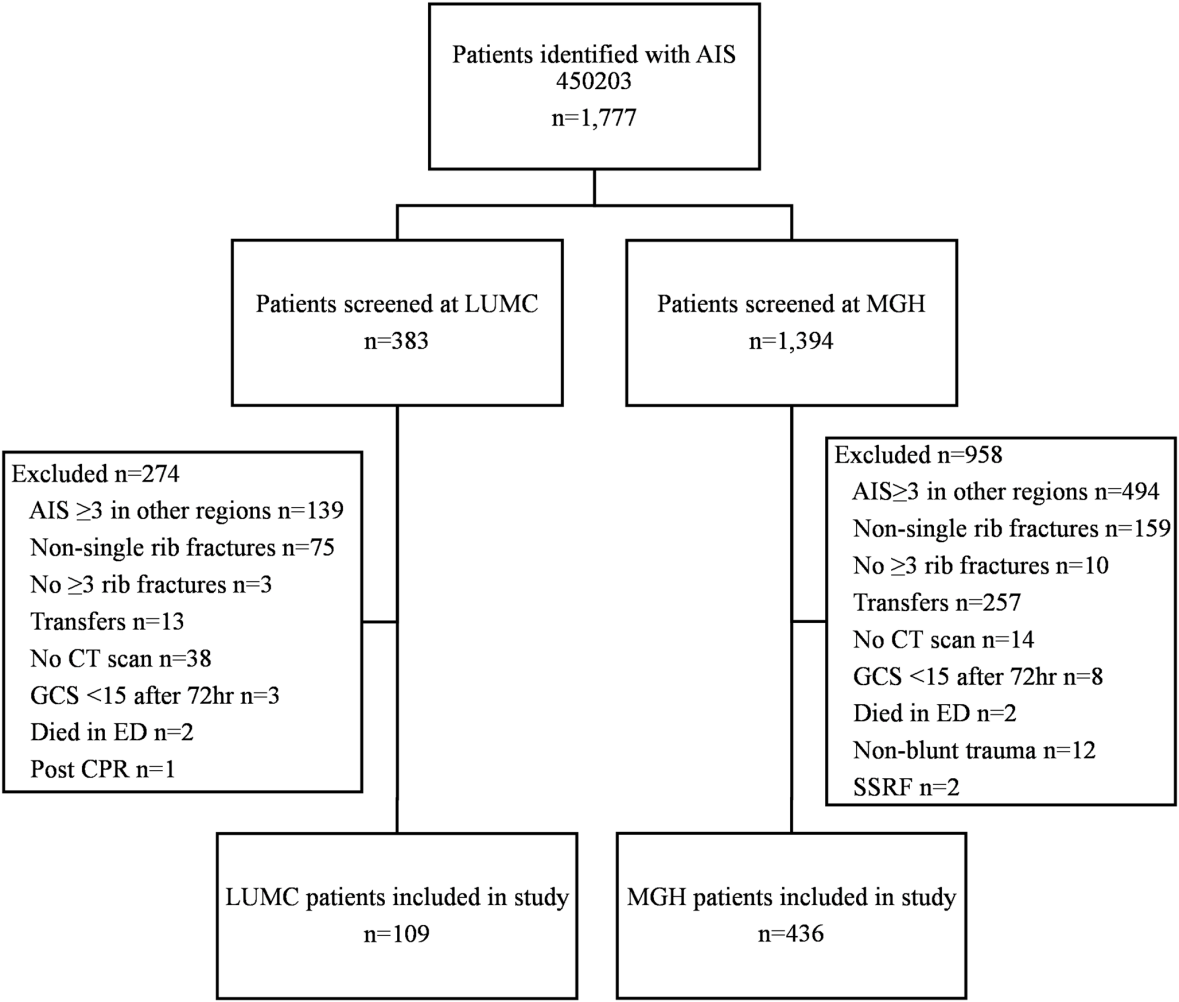
Flowchart of patient inclusion and exclusion at LUMC and MGH

A total of 1,777 patients were identified who were admitted with multiple rib fractures as coded by AIS code 450,203 across both centers, with 383 at LUMC and 1,394 at MGH. After reviewing all patients for the inclusion and exclusion criteria, 545 were included in the final analysis: 109 from LUMC and 436 from MGH. The most common reasons for exclusion were severe injuries in regions other than the thorax and ribs fractured in multiple locations within the same rib (Fig. 1).

### Patient characteristics

Patient demographics and baseline characteristics are summarized in Table 1. MGH patients were older than LUMC patients (median 68 vs. 61, *p* < 0.001), with more patients being over 65 years old (56.7% vs. 39.4%, *p* = 0.001). MGH patients had a higher rate of obesity (BMI ≥ 30: 30.0% vs. 17.4%, *p* = 0.008), a lower rate of smoking (23.6% vs. 33.3%, *p* = 0.045), higher rates of congestive heart failure (CHF) (12.6% vs. 3.7%, *p* = 0.007) and diabetes (21.1% vs. 9.2%, *p* = 0.004), and a borderline higher rate of chronic obstructive pulmonary disease (12.6% vs. 7.3%, *p* = 0.050). Additionally, MGH patients were more likely to use bronchodilators at the time of admission (16.7% vs. 9.2%, *p* = 0.049) and had higher rates of chronic opioid use (13.6% vs. 3.8%, *p* = 0.005).

**Table 1:** Patient demographics and baseline characteristics, stratified by treatment center

	Total *n* = 545	LUMC *n* = 109	MGH *n* = 436	*p*-value
Age in years	66 (53–79)	61 (48–73)	68 (54-80.5)	< 0.001
Age > 65 years	290 (53.2)	43 (39.4)	247 (56.7)	0.001
Female sex	217 (39.7)	41 (37.6)	176 (40.4)	0.60
Obesity (BMI ≥ 30)	150 (27.5)	19 (17.4)	131 (30.0)	0.008
Smoker	135 (25.4)	33 (33.3)	102 (23.6)	0.045
COPD	63 (11.5)	8 (7.3)	55 (12.6)	0.050
Asthma	51 (9.4)	5 (4.6)	46 (10.6)	0.056
CHF	59 (10.8)	4 (3.7)	55 (12.6)	0.007
Diabetes	102 (18.7)	10 (9.2)	92 (21.1)	0.004
Dementia	34 (6.2)	3 (2.8)	31 (7.1)	0.092
Chronic opioid use	56 (11.5)	4 (3.8)	52 (13.6)	0.005
Bronchodilator use	83 (15.2)	10 (9.2)	73 (16.7)	0.049
Inhaled corticosteroid use	28 (5.1)	5 (4.6)	23 (5.3)	0.77
Oral corticosteroid use	26 (4.8)	2 (1.8)	24 (5.5)	0.11

Categorical variables reported as n (%). Continuous variables reported as median (Inter Quartile Range). BMI: body mass index, COPD: chronic obstructive pulmonary disease, CHF: congestive heart failure

### Trauma and fracture characteristics

As shown in Table 2, LUMC patients presented with more severe injuries, reflected in a higher median injury severity score (ISS) (13 vs. 10, *p* < 0.001) and higher thoracic AIS scores, with 11.9% vs. 1.6% having AIS > 3 injuries (*p* < 0.001). LUMC patients had more fractured ribs (median 5 vs. 4, *p* = 0.023), with generally more complex fracture patterns. They more commonly sustained multiregional (≥ 2 anatomical regions) rib fractures (52.3% vs. 32.2%, *p* < 0.001) with higher rates of wedge (21.1% vs. 11.0%) and complex (17.4% vs. 8.7%) fracture types (*p* < 0.001). Consistent with a higher injury severity score, LUMC patients had higher rates of concomitant injuries in the thorax as well as a higher rate of non-severe (AIS < 3) injuries in other body regions (83.5% vs. 70.4%, *p* = 0.006). LUMC patients had higher rates of pneumothorax (33.0% vs. 21.3%, *p* = 0.010) and pulmonary contusion (24.8% vs. 14.0%, *p* = 0.006).

**Table 2:** Trauma and rib fracture characteristics, stratified by treatment center

	Total *n* = 545	LUMC *n* = 109	MGH *n* = 436	*p*-value
Fractured ribs	4 (3–6)	5 (4–6)	4 (3–5)	0.023
Fracture type				< 0.001
Simple	417 (76.5)	67 (61.5)	350 (80.3)	
Wedge	71 (13.0)	23 (21.1)	48 (11.0)	
Complex	57 (10.5)	19 (17.4)	38 (8.7)	
Fracture displacement				0.32
Nondisplaced	216 (39.6)	37 (33.9)	179 (41.1)	
Offset	162 (29.7)	33 (30.3)	129 (29.6)	
Displaced	167 (30.6)	39 (35.8)	128 (29.4)	
Bilateral rib fractures	96 (17.6)	16 (14.7)	80 (18.3)	0.37
Multiregional rib fracture^1^	198 (36.2)	57 (52.3)	141 (32.2)	< 0.001
Pneumothorax	129 (23.7)	36 (33.0)	93 (21.3)	0.010
Hemothorax	70 (12.8)	10 (9.2)	60 (13.8)	0.20
Pulmonary contusion	88 (16.1)	27 (24.8)	61 (14.0)	0.006
Thoracic AIS				< 0.001
3	525 (96.3)	96 (88.1)	429 (98.4)	
4	18 (3.3)	13 (11.9)	5 (1.1)	
5	2 (0.4)	0 (0.0)	2 (0.5)	
Concomitant injury outside the chest^2^	398 (73.0)	91 (83.5)	307 (70.4)	0.006
ISS	11 (9–14)	13 (10–17)	10 (9–14)	< 0.001

Categorical variables reported as n (%). Continuous variables reported as median (Inter Quartile Range). ISS: injury severity score, AIS: last digit of Abbreviated Injury Scale

^1^Multiregional indicates involvement of ≥ 2 anatomical rib regions

^2^Only including injuries with AIS < 3, as concomitant injury with AIS > 3 outside the chest was an exclusion criterion for the study

### Pain management

Pain management approaches differed significantly between centers while achieving similar pain-related outcomes (Table 3). LUMC patients reported lower pain scores at admission (median 6 vs. 8, *p* < 0.001) but showed a similar decrease in pain scores between admission and discharge (median 4 vs. 4, *p* = 0.65). In both LUMC and MGH, oral opioids were used most frequently as strongest initial pain management modality (55.0% vs. 51.4%). Intravenous (IV) Patient-controlled analgesia (PCA) was more frequently chosen as the strongest initial pain management modality at LUMC (22.9% vs. 5.7%), while standard IV opioids (11.0% vs. 21.3%) and lidocaine patches (0.0% vs. 12.4%) were more often applied as the strongest initial modality at MGH. Nerve blocks were used infrequently at both centers (1.8% vs. 5.0%) (*p* < 0.001). In addition to higher PCA use, LUMC patients had longer PC(E)A duration (median 4 (3–6) vs. 3 (2–5) days, *p* = 0.003). Within 72 h of admission, LUMC patients were more likely to have pain management stepped down (21.1% vs. 19.7%, *p* < 0.001) while MGH patients were more than twice as likely to have treatment stepped up (12.4% vs. 5.5%, *p* < 0.001). Despite these differences, patients were discharged with similar opioid requirements (median OME 45 vs. 45, *p* = 0.75). Among patients with no history of chronic opioid use, 78.3% of LUMC patients compared to 69.9% of MGH patients were prescribed opioids at discharge (*p* = 0.085), with comparable dosages among these new users (median OME 60 vs. 60, *p* = 0.78).

**Table 3:** Pain management strategies and effectiveness, stratified by treatment center

	Total *n* = 545	LUMC *n* = 109	MGH *n* = 436	*p*-value
Admission location				0.78
Ward	450 (82.6)	89 (81.7)	361 (82.8)	
ICU	95 (17.4)	20 (18.3)	75 (17.2)	
Admission pain score	8 (6–9)	6 (5–8)	8 (6–10)	< 0.001
Decrease in pain score (initial - discharge)	4 (2–5)	4 (2–5)	4 (2–6)	0.68
Initial pain management modality (strongest applied treatment)^1^				0.002
No medication	3 (0.6)	0 (0.0)	3 (0.7)	
Oral non-opioid	25 (4.6)	10 (9.2)	15 (3.4)	
Lidocaine patch	54 (9.9)	0 (0.0)	54 (12.4)	
Oral opioid	284 (52.1)	60 (55.0)	224 (51.4)	
IV opioid	105 (19.3)	12 (11.0)	93 (21.3)	
IV PCA	50 (9.2)	25 (22.9)	25 (5.7)	
Block (nerve block or epidural)	24 (4.4)	2 (1.8)	22 (5.0)	
Pain management change within 72 h				< 0.001
No change	337 (61.8)	57 (52.3)	280 (64.2)	
Step-up	60 (11.0)	6 (5.5)	54 (12.4)	
Step-down	109 (20.0)	23 (21.1)	86 (19.7)	
PC(E)A duration	3 (2–5)	4 (3–6)	3 (2–5)	0.003
OME at discharge (all)	45 (30–85)	45 (30–70)	45 (30–90)	0.75
New opioid use at discharge	382 (71.5)	83 (78.3)	299 (69.9)	0.085
OME at discharge (new use only)	60 (40–90)	60 (40–75)	60 (30–90)	0.78

Categorical variables reported as n (%). Continuous variables reported as median (Inter Quartile Range). IV: intravenous, PC(E)A: patient controlled (epidural) analgesia, OME: oral morphine equivalents

^1^Each patient was categorized based on the strongest initial pain intervention received, in ascending order of intensity: no analgesia, oral non-opioid, lidocaine patch, oral opioid, IV opioid, IV PCA, regional block/epidural

### Hospital course and clinical outcomes

Hospital course and clinical outcomes were comparable between treatment centers despite the higher trauma burden seen in LUMC patients, as outlined in Table 4. HLOS was similar between centers (median 3 (1–7) vs. 4 (2–6) days, *p* = 0.059). Although taken to the ICU at a similar rate (18.3% vs. 18.6%, *p* = 0.96), LUMC patients had significantly shorter ICU LOS (median 1 (1–2) vs. 3 (1–5) days, *p* < 0.001) compared to MGH patients. Only 11 patients (2.0%) required mechanical ventilation, at MGH. Complications were rare and the rates were similar in both centers, with pulmonary complications occurring in 3.7% of LUMC patients and 6.2% of MGH patients (*p* = 0.31). Most common complications were pneumonia (1.8% vs. 2.5%, *p* = 0.67) and mortality (2.8% vs. 1.8%, *p* = 0.54). Readmission rates were similar (2.8% vs. 2.5%, *p* = 0.89).

**Table 4:** Hospital course and outcomes, stratified by treatment center

	Total *n* = 545	LUMC *n* = 109	MGH *n* = 436	*p*-value
Hospital LOS	4 (2–6)	3 (1–7)	4 (2–6)	0.059
ICU admission (any)	101 (18.5)	20 (18.3)	81 (18.6)	0.96
ICU LOS	2 (1–5)	1 (1–2)	3 (1–5)	< 0.001
Mechanical ventilation	11 (2.0)	0 (0.0)	11 (2.5)	0.094
Chest tube placement	57 (10.5)	15 (13.8)	42 (9.6)	0.21
Total chest tube duration (days)	3 (2–4)	3 (2–4)	3 (2–5)	0.81
Mortality	11 (2.0)	3 (2.8)	8 (1.8)	0.54
Pneumonia	13 (2.4)	2 (1.8)	11 (2.5)	0.67
Empyema	2 (0.4)	1 (0.9)	1 (0.2)	0.29
In-hospital pneumothorax	7 (1.3)	0 (0.0)	7 (1.6)	0.18
In-hospital hemothorax	7 (1.3)	1 (0.9)	6 (1.4)	0.70
Unplanned intubation	5 (0.9)	0 (0.0)	5 (1.1)	0.26
Pulmonary complication^1^	31 (5.7)	4 (3.7)	27 (6.2)	0.31
Readmission	14 (2.6)	3 (2.8)	11 (2.5)	0.89

Categorical variables reported as n (%). Continuous variables reported as median (Inter Quartile Range). LOS: length of stay, ICU: intensive care unit, SSRF: surgical fixation of rib fractures

^1^Any of the following complications: pneumonia, ventilator-associated pneumonia (VAP), empyema, in-hospital pneumothorax, in-hospital hemothorax, and unplanned intubation

### Predictors of pneumonia

Forward stepwise logistic regression analysis identified two statistically significant, independent predictors of pneumonia: CHF (OR 14.86, 95% CI 3.24–68.16, *p* = 0.001) and chronic obstructive pulmonary disease (OR 8.08, 95% CI 1.72–38.03, *p* = 0.008).

### Subgroup analyses

Patients with bilateral rib fractures (*n* = 96, 17.6%) were more likely to be admitted to the ICU at admission (26.0% vs. 15.6%, *p* = 0.014), but had similar ICU LOS (median 2 (1–4) vs. 2 (1–5) days, *p* = 0.77) and HLOS (median 4.5 (3–7) vs. 4 (2–6) days, *p* = 0.15), compared to patients with unilateral rib fractures. Pulmonary complications occurred at similar rates (4.2% vs. 6.0%, *p* = 0.48), with pneumonia rates of 2.1% vs. 2.4% (*p* = 0.83). Mortality rates were also comparable (2.1% vs. 2.0%, *p* = 0.96) (Table 5).

**Table 5:** Hospital course and outcomes, stratified by fracture location

	Total *n* = 545	Unilateral *n* = 449	Bilateral *n* = 96	*p*-value
Admission location				0.014
Ward	450 (82.6)	379 (84.4)	71 (74.0)	
ICU	95 (17.4)	70 (15.6)	25 (26.0)	
Hospital LOS	4 (2–6)	4 (2–6)	4.5 (3–7)	0.15
ICU admission (any)	101 (18.5)	76 (16.9)	25 (26.0)	0.037
ICU LOS	2 (1–5)	2 (1–5)	2 (1–4)	0.77
Mechanical ventilation	11 (2.0)	9 (2.0)	2 (2.1)	0.96
Chest tube placement	57 (10.5)	52 (11.6)	5 (5.2)	0.064
Total chest tube duration (days)	3 (2–4)	3 (2–4)	4 (2–5)	0.64
Mortality	11 (2.0)	9 (2.0)	2 (2.1)	0.96
Pneumonia	13 (2.4)	11 (2.4)	2 (2.1)	0.83
Empyema	2 (0.4)	2 (0.4)	0 (0.0)	0.51
In-hospital pneumothorax	7 (1.3)	6 (1.3)	1 (1.0)	0.82
In-hospital hemothorax	7 (1.3)	7 (1.6)	0 (0.0)	0.22
Unplanned intubation	5 (0.9)	4 (0.9)	1 (1.0)	0.89
Pulmonary complication^1^	31 (5.7)	27 (6.0)	4 (4.2)	0.48
Readmission	14 (2.6)	11 (2.4)	3 (3.1)	0.70

Categorical variables reported as n (%). Continuous variables reported as median (Inter Quartile Range). LOS: length of stay, ICU: intensive care unit, SSRF: surgical fixation of rib fractures

^1^Any of the following complications: pneumonia, ventilator-associated pneumonia (VAP), empyema, in-hospital pneumothorax, in-hospital hemothorax, and unplanned intubation

Patients with displaced rib fractures (*n* = 167, 30.6%) were also more likely to be admitted to the ICU at admission (27.5% vs. 13.0%, *p* < 0.001), with similar ICU LOS (median 2 (1–4) vs. 2 (1–5) days, *p* = 0.18) and HLOS (median 4 (2–7) vs. 4 (2–6) days, *p* = 0.18). These patients were also more likely to require mechanical ventilation (4.2% vs. 1.1%, *p* = 0.016) and chest tube placement (15.6% vs. 8.2%, *p* = 0.010). While associated with higher rates of in-hospital hemothorax (3.0% vs. 0.5%, *p* = 0.018), overall pulmonary complications (7.2% vs. 5.0%, *p* = 0.32), including pneumonia rates of 1.2% vs. 2.9% (*p* = 0.23) were similar. Mortality rates were also comparable (3.0% vs. 1.6%, *p* = 0.28) (Table 6).

**Table 6:** Hospital course and outcomes, stratified by fracture displacement

	Total *n* = 545	Non-displaced *n* = 378	Displaced *n* = 167	*p*-value
Admission location				< 0.001
Ward	450 (82.6)	329 (87.0)	121 (72.5)	
ICU	95 (17.4)	49 (13.0)	46 (27.5)	
Hospital LOS	4 (2–6)	4 (2–6)	4 (2–7)	0.18
ICU admission (any)	101 (18.5)	54 (14.3)	47 (28.1)	< 0.001
ICU LOS	2 (1–5)	2 (1–5)	2 (1–4)	0.18
Mechanical ventilation	11 (2.0)	4 (1.1)	7 (4.2)	0.016
Chest tube placement	57 (10.5)	31 (8.2)	26 (15.6)	0.010
Total chest tube duration (days)	3 (2–4)	3 (2–4)	3 (2–4)	0.34
Mortality	11 (2.0)	6 (1.6)	5 (3.0)	0.28
Pneumonia	13 (2.4)	11 (2.9)	2 (1.2)	0.23
Empyema	2 (0.4)	1 (0.3)	1 (0.6)	0.55
In-hospital pneumothorax	7 (1.3)	4 (1.1)	3 (1.8)	0.48
In-hospital hemothorax	7 (1.3)	2 (0.5)	5 (3.0)	0.018
Unplanned intubation	5 (0.9)	3 (0.8)	2 (1.2)	0.65
Pulmonary complication^1^	31 (5.7)	19 (5.0)	12 (7.2)	0.32
Readmission	14 (2.6)	7 (1.9)	7 (4.2)	0.11

Categorical variables reported as n (%). Continuous variables reported as median (Inter Quartile Range). LOS: length of stay, ICU: intensive care unit, SSRF: surgical fixation of rib fractures

^1^Any of the following complications: pneumonia, ventilator-associated pneumonia (VAP), empyema, in-hospital pneumothorax, in-hospital hemothorax, and unplanned intubation

Patients requiring ICU admission at any time during hospitalization (*n* = 101, 18.5%) were older (median 74 (61–84) vs. 65 (52–78) years, *p* < 0.001), more frequently had CHF (17.8% vs. 9.2%, *p* = 0.012), and presented with more severe injuries, reflected in higher ISS (median 14 (10–15) vs. 10 (9–14), *p* = 0.002). ICU patients had more fractured ribs (median 5 (3–7) vs. 4 (3–5), *p* = 0.020), with more frequently displaced (46.5% vs. 27.0%, *p* < 0.001) and bilateral fractures (24.8% vs. 16.0%, *p* = 0.037), as well as higher rates of pulmonary contusion (27.7% vs. 13.5%, *p* < 0.001).

Most ICU patients (93.1%) were admitted directly to the ICU, while only 6.9% required ICU escalation during hospitalization. Although initial pain scores at admission were comparable between ICU and non-ICU patients (median 7 (7–8) vs. 8 (7–10), *p* = 0.096), pain management nevertheless differed between these groups. ICU patients more frequently received stronger initial pain management modalities including IV opioids (25.7% vs. 17.8%), IV PCA (21.8% vs. 6.3%) and regional blocks (7.9% vs. 3.6%), while non-ICU patients more often received oral opioids (56.5% vs. 32.7%) (*p* < 0.001). When given PCA, ICU patients had longer PCA duration (median 4 (2–6) vs. 3 (2–4) days, *p* = 0.021). Additionally, ICU patients more frequently required a step-up in pain management within 72 h (18.9% vs. 10.2%, *p* = 0.040).

ICU patients had significantly longer hospital stays (median 7 (5–13) vs. 3 (2–6) days, *p* < 0.001) compared to non-ICU patients. ICU patients experienced more pulmonary complications (18.8% vs. 2.7%, *p* < 0.001), including pneumonia (8.9% vs. 0.9%, *p* < 0.001). Chest tube placement rates were similar between groups (14.9% vs. 9.5%, *p* = 0.06), though ICU patients had a chest tube for a slightly longer period (median 3.5 (3-9.5) vs. 3 (2–4) days, *p* = 0.009). In-hospital hemothorax occurred more frequently in ICU patients (5.0% vs. 0.5%, *p* < 0.001), while in-hospital pneumothorax rates were comparable (2.0% vs. 1.1%, *p* = 0.49). Mortality was significantly higher among ICU patients (7.9% vs. 0.7%, *p* < 0.001), as were readmission rates (6.9% vs. 1.6%, *p* = 0.002) (Table 7).

**Table 7:** Hospital course and outcomes, stratified by ICU admission (any)

	Total *n* = 545	Non-ICU *n* = 444	ICU *n* = 101	*p*-value
Admission location				
Ward	450 (82.6)	444 (100)	7 (6.9)	
ICU	95 (17.4)		94 (93.1)	
ICU LOS			2 (1–5)	
Mechanical ventilation			11 (10.9)	
Chest tube placement	57 (10.5)	42 (9.5)	15 (14.9)	0.06
Total chest tube duration (days)	3 (2–4)	3 (2–4)	3.5 (3-9.5)	0.009
Mortality	11 (2.0)	3 (0.7)	8 (7.9)	< 0.001
Pneumonia	13 (2.4)	4 (0.9)	9 (8.9)	< 0.001
Empyema	2 (0.4)	1 (0.2)	1 (1.0)	0.251
In-hospital pneumothorax	7 (1.3)	5 (1.1)	2 (2.0)	0.491
In-hospital hemothorax	7 (1.3)	2 (0.5)	5 (5.0)	< 0.001
Unplanned intubation	5 (0.9)	0 (0.0)	5 (5.0)	< 0.001
Pulmonary complication^1^	31 (5.7)	12 (2.7)	19 (18.8)	< 0.001
Readmission	14 (2.6)	7 (1.6)	7 (6.9)	0.002
Hospital LOS	4 (2–6)	3 (2–6)	7 (5–13)	< 0.001

Categorical variables reported as n (%). Continuous variables reported as median (Inter Quartile Range). LOS: length of stay, ICU: intensive care unit, SSRF: surgical fixation of rib fractures

^1^Any of the following complications: pneumonia, ventilator-associated pneumonia (VAP), empyema, in-hospital pneumothorax, in-hospital hemothorax, and unplanned intubation

## Discussion

This international comparison between two level 1 trauma centers compared pain management approaches and clinical outcomes for patients with multiple single rib fractures who were treated non-operatively. Despite significant baseline differences in patient characteristics, injury severity and pain management strategies, clinical outcomes remained comparable between centers, with similar hospital length of stay, mortality, opioid prescriptions at discharge and rates of pulmonary complications on univariate analysis. CHF and COPD were identified as predictors of pneumonia development, consistent with previously reported risk factors for worse outcomes in rib fracture patients [21, 22]. 

LUMC patients were significantly younger and had fewer comorbidities than MGH patients. However, they presented with more severe injuries, reflected in higher ISS, more concomitant injuries, and more complex fracture patterns. These differences may reflect variations in trauma patterns, referral practices or pre-hospital care between geographical regions. Despite baseline and injury severity differences, both centers showed similar clinical outcomes, with comparable hospital length of stay, complication rates, and mortality.

Although similar effectiveness of pain management, measured by pain score change and OME prescribed at discharge, was achieved, the two centers differed significantly in their pain management strategies. Oral opioid use was used predominantly in both centers, but when stepped up to IV administration, the LUMC favored the use of PCA while MGH more frequently used standard IV opioids. For non-opioid management, MGH often utilized lidocaine patches, while LUMC primarily used oral non-opioids. Pain management also evolved differently, with the Dutch center more commonly stepping down in pain management by discontinuing and reducing analgesic intensity, while the US center more often stepped up by adding medication or changing to more intensive strategies. This variation in pain management may reflect concerns about opioid use and complications in the US healthcare system compared to Western Europe [23], but may also reflect varying underlying pain management philosophies, such as differences in patient autonomy regarding pain control, risk tolerance for opioid-related adverse outcomes, and cultural beliefs toward pain presentation and management [24]. These differences in philosophies may explain MGH’s preference for lidocaine patches adjunctive to oral non-opioids, employing multimodal non-opioid pain management, and their use of standard IV opioids over PCA, reflecting a preference for provider-controlled rather than patient-controlled administration. Despite higher rates of chronic opioid use among MGH patients at baseline, both centers prescribed similar opioid dosages at discharge. Among patients with no history of chronic opioid use, MGH showed a slightly lower, though not statistically significant, proportion of newly prescribed opioids at discharge. This finding contrasts with previous literature suggesting more conservative opioid prescribing practices in Western Europe compared to the US [9–11]. These findings suggest that institutional heterogeneity in pain management can achieve similar clinical outcomes, implying the effectiveness of multiple treatment models rather than requiring standardization in rigid guidelines.

Subgroup analyses showed that patients with more severe rib fracture patterns required more intensive treatment without necessarily experiencing worse clinical outcomes. Both patients with bilateral rib fractures and displaced fractures were more likely to require ICU admission. Patients with displaced fractures also more frequently required mechanical ventilation and chest tube placement compared to patients with nondisplaced fractures. However, complication rates and mortality remained comparable to patients with unilateral or nondisplaced fractures, apart from higher rates of in-hospital hemothorax among patients with displaced fractures. Bilateral and displaced fractures are regarded as predictors of complications and are incorporated in risk scores such as the RibScore [25]. However, in this cohort these fracture patterns primarily resulted in more intensive treatment rather than necessarily leading to worse outcomes.

The ICU subgroup provides insight into high-risk patients with multiple single rib fractures who might require more intensive treatment. ICU patients were characterized by older age, higher comorbidity, and more severe injury patterns, including more pulmonary contusion, fractured ribs, bilateral and displaced fractures. Despite receiving more intensive pain management through IV PCA and regional block use, ICU patients experienced substantially worse outcomes, such as higher rates of pulmonary complications, longer hospital stays, and increased mortality. These findings demonstrate that patients with more complex fracture patterns, such as bilateral and displaced fractures, experience worse outcomes despite intensive treatment, emphasizing the need for close monitoring of this high-risk group. Given the older age, higher comorbidity, and more severe injury patterns in ICU patients, the worse outcomes likely reflect baseline patient characteristics and their injury severity, highlighting the importance of early recognition and close monitoring of this vulnerable population.

This study has several limitations that should be considered when interpreting these findings. First, the single-center comparison per country limits generalizability and may not represent regional or national treatment patterns. The observed differences in pain management strategies between LUMC and MGH could reflect broader healthcare system practices, but could also be driven by local expertise, resource availability, and institutional preferences. Multi-center studies within each country would be needed to differentiate between institutional variation and true national differences in treatment philosophies. Second, the retrospective study design does not allow for establishing causal associations, which is further complicated by the international comparison design that introduces additional cultural, economic, and systemic confounding factors that further limit causal conclusions. Third, by only including admitted patients, those discharged from the emergency department or treated in an outpatient setting were outside the scope of this study. While this may have introduced some selection bias, the extent to which this influenced the observed differences in patient characteristics and trauma severity between centers cannot be determined. Fourth, this study focused exclusively on pain management strategies, while comprehensive non-operative treatment of rib fractures includes other critical elements including early mobilization, pulmonary hygiene, and physical therapy. Differences in these unmeasured elements of care may have influenced outcomes. Fifth, cultural variations in pain perception and reporting may have influenced subjective patient-reported outcomes, such as pain scores [24], which could explain the observed difference in baseline pain score between centers. Finally, statistical power was limited due to low event rates for many outcome measures. The wide confidence intervals observed in the stepwise logistic regression reflect this limited power and indicate uncertainty in effect estimates. As a result, the study’s ability to detect smaller differences in outcomes was limited, and clinically relevant differences may have gone undetected.

This international comparison shows that despite significant differences in patient and trauma characteristics and pain management strategies, clinical outcomes for multiple single rib fractures were similar between treatment centers. This suggests that similar outcomes can be achieved through different systematic approaches to pain management, advocating for consistent institutional practices and calling into question the need for rigid standardized guidelines. Future research should focus on identifying which institutional factors and systematic approaches improve outcomes for patients with multiple single rib fractures. Additionally, the high-risk ICU population identified in this study represents an important target for research aimed at optimizing pain management strategies and improving outcomes in this vulnerable population.

## Data Availability

The datasets generated during this study are not available due to institutional data sharing restrictions and patient privacy protection regulations.
